# Effect of Mn on the Properties of Powder Metallurgy Ti-2.5Al-xMn Alloys

**DOI:** 10.3390/ma16144917

**Published:** 2023-07-10

**Authors:** Yousef Alshammari, Shaira Mendoza, Fei Yang, Leandro Bolzoni

**Affiliations:** 1 School of Engineering, The University of Waikato, Private Bag 3105, Hamilton 3240, New Zealand; 2 College of Engineering, International University of Science and Technology in Kuwait, Mohamad Bin Qasim Street, Ardiya 92400, Kuwait

**Keywords:** titanium alloys, powder metallurgy, blended elemental, homogeneous microstructure, mechanical properties

## Abstract

Titanium alloys are the ideal material for a wide range of structural applications, but their high cost compared to other metals hinders their adoption. Powder metallurgy and cheap alloying elements can be used to create new Ti alloys. In this study, the simultaneous addition of Al and Mn is considered to manufacture and characterise ternary Ti-2.5Al-Mn alloys obtained via pressing and sintering by varying the Mn content (1–10 wt.%). It is found that the addition of the alloying elements reduces compressibility. Consequently, the amount of porosity increases (8.5 → 10.8%) with the amount of Mn as the alloys were processed under the same conditions. The progressive addition of Mn refines the classical lamellar microstructure and, eventually, transforms it into an equiaxed β-grain structure with acicular α grains. The microstructural changes lead to continuous increases in strength (ultimate tensile strength: 694 → 851 MPa) and hardness (225 → 325 HV30) with an associated loss of ductility (elongation to failure: 13.9 → 1.0%). However, the obtained ternary Ti-2.5Al-Mn alloys have similar or better overall mechanical behaviour than most of the binary Ti-Mn alloys obtained through a variety of manufacturing methods.

## 1. Introduction

The unique combination of a good balance of mechanical properties (i.e., low density and high strength), corrosion resistance, and biocompatibility makes titanium (Ti) alloys one of the best classes of materials for their use in a wide range of engineering applications [1,2,3]. However, a major limitation of Ti alloys is their cost with respect to other structural metals. Powder metallurgy enables manufacturers to achieve a significant reduction in the production costs of Ti alloys. It is, therefore, the ideal technique to develop and manufacture Ti alloys [4,5]. The major advantages include solid-state processing (i.e., reduced reactivity with processing tools), net-shape capability, freedom in selecting alloying elements, and limited amount of machining required [6,7]. The combination of properties achievable in Ti alloys derives from the modification of pure Ti with specific alloying elements, which generally either stabilise the low-temperature α or the high-temperature β phase [8,9].

Amongst the different available alloying elements, aluminium (Al) is the primary α-stabilising element intentionally added to Ti to enhance its mechanical behaviour through the formation of a dual-phase region. Nonetheless, Al is rarely used as the only alloying element, and it is added in combination with β stabilisers to achieve a better balance between strength and ductility [10]. Although not extensively used in wrought Ti alloys, manganese (Mn) is a strong β stabiliser which brings significant strengthening and burn resistance [11] like in the case of the Ti-8Mn alloy.

Mn has lately been more broadly considered for the development of binary Ti-Mn alloys. As commodity and widely available metals, the simultaneous addition of Al and Mn can be used to create innovative Ti alloys.

Examples of studies on binary Ti-(5-20)Mn alloys (% refers to mass unless otherwise indicated) obtained via casting are the work of Gouda et al. [12] and Kim et al. [13]. Gouda et al. [12] used a mixture of pure Ti sponge and Mn flakes in an electric arc furnace under an inert atmosphere to obtain Ti-(8-20)Mn alloys. The alloys were subsequently solution treated (900 °C/10 min), followed by quenching in ice water. The main conclusions of the study are that the cold workability increases with the amount of Mn if limited to 16%, and that the Mn content has limited effect on microhardness (HV_0.5_). Using Ti sponge and Mn ingots, Kim et al. [13] manufactured Ti-(5-20)Mn alloys via arc melting on a water-cooled copper hearth using a tungsten electrode under a high-purity argon atmosphere. The alloys were also heat treated under an argon atmosphere for 4 h at a temperature 150 °C lower than their respective solidus temperature before cooling through a combination of furnace and air cooling. The main finding was that the cast Ti-(5-20)Mn alloys exhibit higher hardness, better oxidation protection, and better corrosion resistance than pure Ti. A major example of binary Ti-(8-17)Mn alloys developed through powder metallurgy is the work of Fernandes Santos et al. [14]. They used spherical Ti and fine Mn powders for vacuum sintering (1100 °C/8 h) samples shaped via metal injection moulding. The samples were eventually solution treated at 900 °C for 1 h. Fernandes Santos et al. [14] concluded that the tensile strength and hardness of the sintered alloys are comparable to that of alloys fabricated via cold crucible levitation melting if the Mn content is limited to 13%. Following this study, Cho et al. [15] analysed the improvement in strength of the Ti-13Mn alloy through cold rolling. The improvement was justified by the increased dislocation density, finer grain size, lower porosity, and formation of a deformation-induced ω phase.

The combined addition of Al and Mn was used to develop the wrought OT4-1 alloy (Russian grade), which is primarily used for room-temperature structural applications. Changes in its chemical composition during its smelting in a vacuum induction furnace were analysed by Blacha et al. [16]. The superplastic deformation behaviour and microstructure evolution of Ti-2.5Al-1.8Mn alloys were studied by Mikhaylovskaya et al. [17]. Murthy and Sundaresan [18] quantified the fracture toughness of wrought a Ti-Al-Mn alloy subjected to different welding and heat treatment procedures.

In summary, Al is used as a complementary alloying element to strike a good balance of mechanical properties and Mn was used to develop binary alloys. However, the concurrent addition of Al and Mn has not been extensively exploited. This is especially in the case of powder metallurgy as, to the best of the authors’ knowledge, there is no study in the literature analysing the development and characterisation of powder metallurgy ternary Ti-Al-Mn alloys aside from the study by Cai et al. [19]. In that study, Ti-9Al-xMn alloys (x = 0, 1, 2, 4, 6%) were manufactured via spark plasma sintering of high-energy ball-milled elemental powders to quantify electrical resistivity and microhardness. No structural properties like tensile or compression behaviour were reported. Therefore, the aim of this study is to analyse the manufacturing of ternary Ti-Al-Mn alloys via powder metallurgy and characterise their structural behaviour in relation to the changes in microstructure and physical properties brought about by a progressively higher amount of Mn.

## 2. Materials and Methods

**Figure 1:**
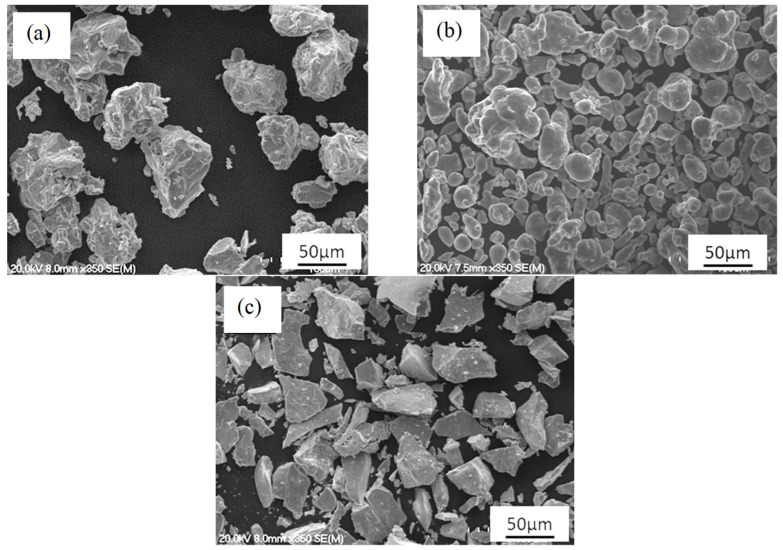
SEM micrographs of the raw materials: (**a**) Ti, (**b**) Al, and (**c**) Mn.

Ternary Ti-Al-Mn alloys were prepared via vacuum sintering of compacted powders. Compositions with a fixed content of the α-stabiliser (2.5% Al) and varying concentrations of the β-stabiliser (1, 5, and 10% Mn) were prepared. The raw powders used for this study were commercial Ti, Al, and Mn powders. The relevant properties of the powders and their morphology are summarised in Table 1 and Figure 1, respectively. As shown in Table 1, the maximum particle size of the alloying elements, 45 μm for Al and 63 μm for Mn, is smaller than that of Ti (75 μm), which should favour the densification of the obtained alloys.

The SEM micrographs in Figure 1 show that Ti and Mn particles have an irregular morphology, whereas Al particles are spherically shaped. The particle morphology of Ti and Mn is ideal for their processing via powder metallurgy as it will lead to a significant number of particles interlocking upon pressing. The spherical morphology of Al is less suited, but its content is relatively low, and Al is highly deformable. Therefore, it is expected that Al powder particles will sit in the gap left by Ti powder particles and plastically deform during shaping of the powder blends.

The ternary compositions were labelled as Ti-2.5Al-1Mn, Ti-2.5Al-5Mn, and Ti-2.5Al-10Mn. It is worth mentioning that the amounts of Al and Mn were, respectively, based on the literature [17] and the authors’ previous work [20]. The powder blends were prepared by mixing at 30 Hz for 30 min in a V-shaped blender (Jiangyin Rongde Machinery, Jiangyin, China) the correct proportions of the raw powders to obtain a homogeneous mixture. The mixture was then compacted into a 40 mm diameter cylindrical sample at room temperature by applying 600 MPa of uniaxial pressure. The resultant green compacts were vacuum sintered at 1250 °C for 2 h in a vacuum furnace (ZSJ − 20 × 20 × 30). A heating rate of 10 °C/min and furnace cooling were used. The selected pressing and sintering conditions were based on the literature available on Ti alloys processed via powder metallurgy [9,21,22,23].

Microstructural characterisation was conducted on polished and etched (Kroll solution, 2 mL of hydrofluoric acid, 6 mL of nitric acid, and 92 mL of distilled water) samples using an Olympus BX 60 optical microscope (Olympus, Auckland, New Zealand). X-ray diffraction (XRD) patterns for the developed compositions were obtained using a scanning rate of 0.013° within the 30° to 80° diffraction angle (Philips X’pert diffractometer, Philips, Amsterdam, The Netherlands).

The rule of mixtures, the mass to volume ratio, and Archimedes’ principle measurements were, respectively, employed to calculate the theoretical, green, and sintered density of the alloys. These data were then used to calculate the densification parameter [(sintered density − green density)/(theoretical density − green density)].

To determine the tensile properties, at least three specimens for each alloy were machined into dog-bone samples via electrical discharge machining. All samples had a gauge length of 20 mm and a rectangular cross-section of 2 × 2 mm^2^. The tensile tests were conducted using a crosshead speed of 0.1 mm/min on an Instron 33R4204 universal testing machine. A mechanical extensometer was used to record the change in elongation. The offset method was chosen to define the yield stress (YS) of the alloys. The average hardness values of the Ti-2.5Al-Mn alloys were calculated using at least five Vickers hardness (HV30) measurements.

## 3. Results

**Figure 2:**
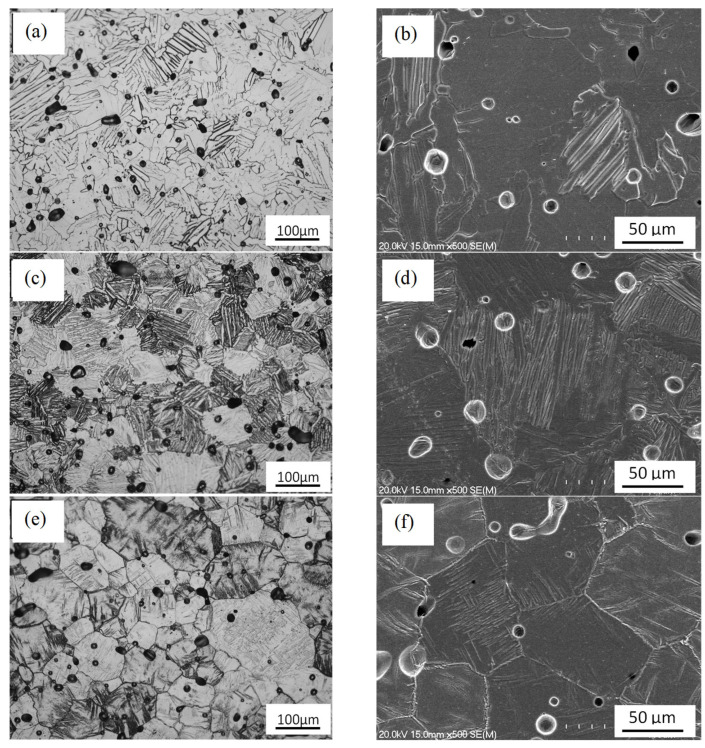
Micrographs (optical and SEM, respectively) of the sintered ternary alloys: (**a**,**b**) Ti-2.5Al-1Mn, (**c**,**d**) Ti-2.5Al-5Mn, and (**e**,**f**) Ti-2.5Al-10Mn.

From the results of the microstructural analysis shown in Figure 2, it can be seen that pores with spherical morphology are found in the microstructure independently of the chemical composition. The presence of residual pores in the microstructure is common for powder metallurgy Ti alloys [24,25,26].

The pore size varies from 18 μm to 80 μm, with smaller pores less common in the samples with a higher Mn content. Thus, an increase in the content of the β-stabilising element in the alloy leads to a gradual increase in both the number of pores and their size.

From the microstructural analysis, it is also found that an increase in the Mn content from 1% (Figure 2a) to 5% (Figure 2c) is accompanied by the formation of a more refined lamellar microstructure and a reduction in the interlamellar spacing (Figure 2b–d). This lamellar microstructure is commonly found in α + β Ti alloys slowly cooled from high temperatures [27,28,29]. With respect to the Ti-2.5Al-10Mn alloys (Figure 2e), its microstructure is characterised by equiaxed β-phase grains and a fine needle-like α lamellae precipitated at the β grain boundaries (Figure 2f). As could be expected based on the current literature, the alloys studied are characterised by a homogeneous microstructure due to the high diffusivity of Al and Mn. The semi-quantitative EDS analysis of the Ti-2.5Al-1Mn, Ti-2.5Al-5Mn, and Ti-2.5Al-10Mn alloys, respectively, yielded Al/Mn contents of 2.34 ± 0.17/1.09 ± 0.10, 2.59 ± 0.08/5.31 ± 0.05, and 2.44 ± 0.09/9.88 ± 0.13 with Ti being the balance.

**Figure 3:**
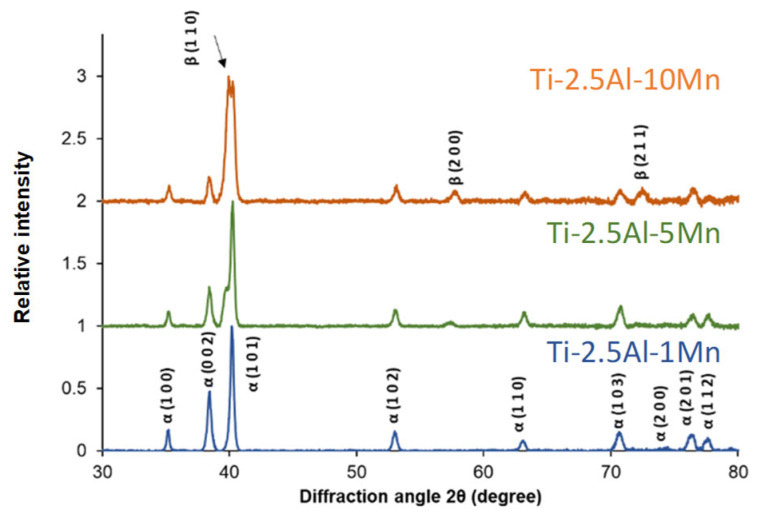
XRD patterns of the sintered ternary alloys.

The XRD pattern of the Ti-2.5Al-1Mn alloy (Figure 3) shows only peaks identified as the α phase, without other crystalline structures, meaning that the amount of stabilised β phase is below the detection limit of the equipment. The increase in the amount of Mn to 5% leads to the emergence of the main β phase’s peak due to the stabilisation of a greater amount of β phase in the microstructure (Figure 2c,d). As the content of Mn is increased to 10%, the relative intensity of the β-phase (110) peak is predominant. Other β-phase peaks are also detected as a consequence of the equiaxed microstructure (Figure 2e).

**Figure 4:**
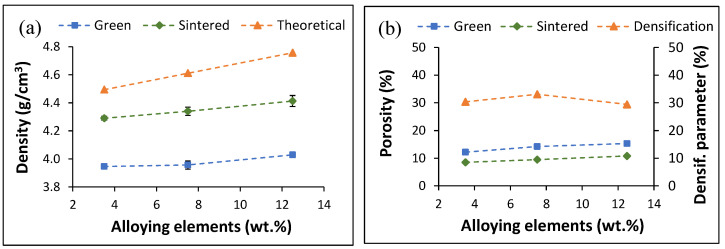
Physical properties of the sintered ternary alloys: (**a**) density and (**b**) porosity/densification parameter.

The variation in the physical properties of the alloys is shown in Figure 4, where it can be seen that the green (3.95 → 4.03 g/cm^3^), sintered (4.29 → 4.41 g/cm^3^), and theoretical (4.49 → 4.76 g/cm^3^) density of the alloys increase with the amounts of alloying elements as a consequence of the relative value of the density of these elements.

In terms of porosity, the amount of pores present in both the green (12.2 → 15.3%) and sintered (8.5 → 10.8%) samples increases as the amount of alloying elements increases. This is due to their specific effects on the compressibility and sinterability of the alloys. Because of that, the densification initially increases but subsequently decreases as more alloying elements are added [30].

**Figure 5:**
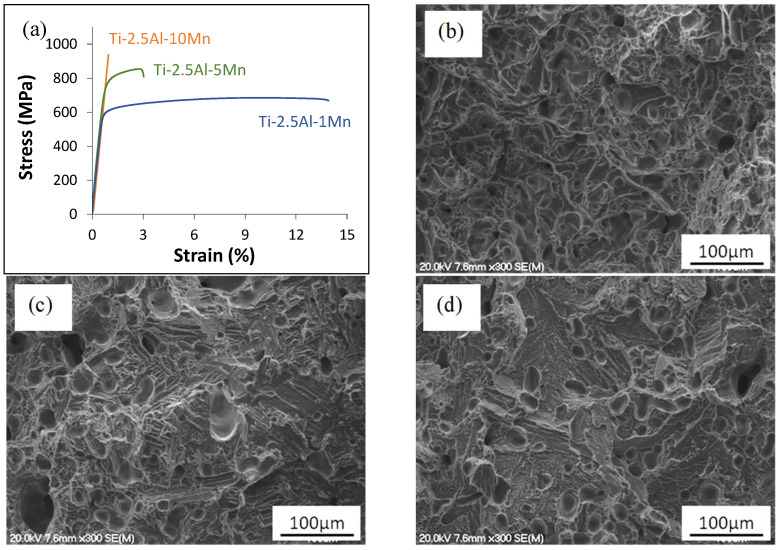
Tensile and fracture behaviour of the sintered ternary alloys: (**a**) representative stress–strain curves, (**b**) fracture surface of Ti-2.5Al-1Mn alloy, (**c**) fracture surface of Ti-2.5Al-5Mn alloy, and (**d**) fracture surface of Ti-2.5Al-10Mn alloy.

As shown in Figure 5, the sintered samples containing 1% and 5% of Mn exhibit ductile behaviour prior to non-catastrophic failure as evidenced by the representative stress–strain curves. The alloy with the highest Mn content (i.e., 10%) demonstrates purely elastic behaviour without plastic deformation.

The three alloys have comparable stiffness (i.e., 100 ± 10 GPa) as their stress–strain curves overlap in the elastic region. As a consequence of their response to the applied uniaxial tensile load, the fracture surface of the Ti-2.5Al-1Mn alloy is composed of ductile dimples (Figure 5b). As the Mn content increases, a small number of transgranularly failed brittle areas start to form, as confirmed by the fractographic analysis of the Ti-2.5Al-5Mn alloy (Figure 5c). Due to its brittle behaviour, the fracture surface of the Ti-2.5Al-10Mn alloy is primarily characterised by the presence of cleavage facets and tear ridges, even though a small number of ductile dimples is still present (Figure 5d).

**Figure 6:**
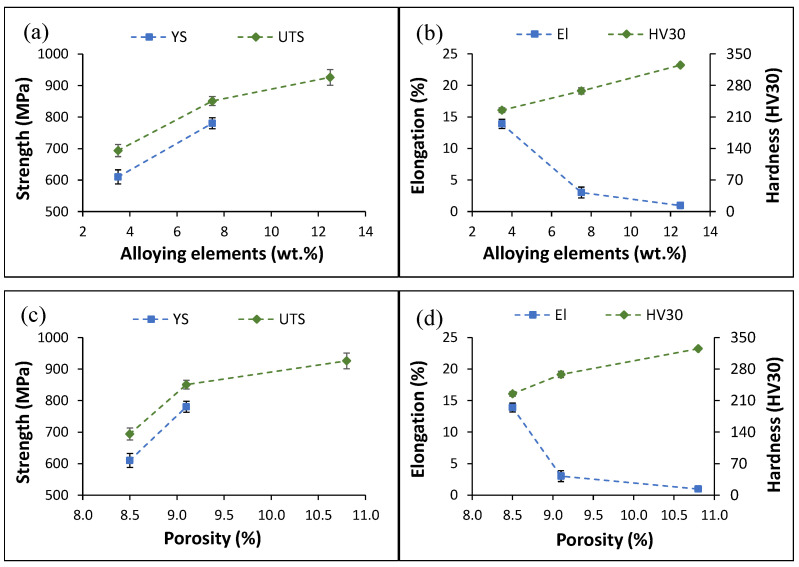
Mean mechanical properties of the sintered ternary alloys: (**a**) strength vs. amount of alloying elements, (**b**) strain/hardness vs. amount of alloying elements, (**c**) strength vs. porosity, and (**d**) strain/hardness vs. porosity.

Consistent with their stress–strain curves, Figure 6 shows that the mean YS and ultimate tensile strength (UTS) progressively increase with the amount of the alloying elements added. However, it can be observed that there is more substantial increase in the ability to withstand the applied tensile load when the Mn content is increased from 1% (YS = 610 ± 22 MPa, and UTS = 694 ± 19 MPa) to 5% (YS = 780 ± 18 MPa, and UTS = 851 ± 14 MPa). A less pronounced increment when the Mn content in increased to 10% (UTS = 926 ± 25 MPa) is found. Due to its brittle behaviour, the Ti-2.5Al-10Mn alloy does not have a YS value. The counterpart of this behaviour is that the elongation to failure sharply decreases with the initial increment in the amount of Mn added (13.9 ± 0.7% → 3.0 ± 0.9%). Further addition of Mn leads to a less significant loss of ductility (1.0 ± 0.1%).

In terms of hardness (Figure 6b), the increment in the Mn content leads to progressively higher hardness (225 → 325 HV30).

The analysis of the variation in the mean mechanical properties against the amount of porosity present in the alloys yields similar trends. The values of YS, UTS, and hardness increase whilst the elongation to failure decreases with an increment in the amount of residual pores. It is, therefore, deduced that the presence of porosity affects the ductility of the alloys much more significantly than their strength (Figure 6c,d).

## 4. Discussion

In this study, a series of ternary Ti alloys bearing Al and Mn as the alloying elements were obtained through the blended elemental powder metallurgy approach. Preparation and processing of the powder blends via the addition of elemental powders (Figure 1) leads to a progressive increase in the density value, regardless of whether it is the green, sintered, or theoretical density. In terms of theoretical density, as Al has a lower density than Ti but Mn has a higher density with respect to both elements, the Ti-2.5Al-1Mn alloy has a lower theoretical density than pure Ti (i.e., 4.51 g/cm^3^). The other ternary alloys have a higher density compared to Ti due to the greater amount of Mn added. Although the green and sintered densities increase with the amount of alloying elements (Figure 4a), it is also found that the amount of residual porosity increases (Figure 4b). With regard to the green samples, this means that the addition of the alloying element powder particles decreases the compressibility of the powder blends. This is due to the higher hardness of Mn and the spherical particle size of the Al powder used. With respect to the amount of porosity left in the sintered alloys, an increasing trend is found. However, it is worth noticing that the gap between the green and sintered porosity initially increases and then decreases as a consequence of the difference in densification. This behaviour is the compromise between the higher drop in compressibility but higher sinterability achieved when the Mn content increases from 1% to 5%, rather than because of the higher additions of Mn.

Thus, the initial increment in Mn increases sinterability whilst a further increase reduces it. This is primarily due to the amount of thermal energy that needs to be invested for the dissolution and homogenisation of the alloying elements. Examples of similar behaviour where the addition of Mn leads to an increment in sintered density and comparable values of residual porosity in sintered Mn-bearing Ti alloys are available in the literature [14,31].

The microstructural analysis shows that the processing conditions used ensure the complete dissolution of the alloying element powder particles. Homogenous chemistry and spherical residual porosity are, therefore, achieved. Complete homogeneity and isolated round pores are typical of the last stage of sintering of blended elemental powder metallurgy Ti-based alloys. The addition of Al and Mn to Ti generally leads to the formation of a lamellar microstructure composed of α grains and α + β lamellae. On average, the size of α grains (i.e., prior β grains) is not significantly affected by the chemical composition. However, the characteristics of the α + β lamellae are (Figure 2). Specifically, the higher the amount of Mn, which is a strong β stabiliser, the greater the amount of stabilised β phase. This results in an overall refined microstructure with finer α lamellae, coarser β lamellae, and smaller interlamellar spacing. The addition of 10% Mn as the β stabiliser is powerful enough to enable the formation of a microstructure composed of equiaxed β grains. However, the precipitation of needle-like α grains primarily at the grain boundaries still occurs (Figure 2e,f). The stabilisation of the β phase was confirmed by the results of the XRD analysis (Figure 3). The relative intensity of the primary β-phase peak increases with the amount of Mn up to the point that it is stronger than that of the α phase in the Ti-2.5Al-10Mn alloy. From the microstructural analysis, it is found that the Ti-2.5Al-1Mn and Ti-2.5Al-5Mn alloys are α + β Ti alloys, whereas the Ti-2.5Al-10Mn alloy is a metastable β alloy under slow cooling conditions [32].

The microstructural changes occurring in the alloys with an increase in the Mn content have a substantial effect on the mechanical properties. The experimental data show a linear increase in the strength and hardness values when increasing the amounts of alloying elements. Consequently, ductility decreases proportionally (Figure 6). Both the Ti-2.5Al-1Mn and Ti-2.5Al-5Mn alloys exhibit ductile behaviour, whilst the Ti-2.5Al-10Mn alloy shows only elastic behaviour (Figure 5) due to the presence of needle-like precipitated α grains (Figure 2). Accordingly, the fracture surface switches from purely ductile composed of dimples with low additions of Mn to a more brittle surface. The latter is composed of cleavage facets due to the transgranular failure of the α + β lamellae and tear ridges as the Mn content increases (Figure 5). The initial increment in the Mn content leads to significant strengthening of the alloys through the stabilisation of a greater amount of β phase, the consequent refinement of the features of the lamellar microstructure, and the solid solution. These three factors overcome the negative effect of having a higher amount of residual porosity. The same is true of higher addition rates of Mn. However, the transition to a microstructure composed of equiaxed β grains with needle-like α grains remarkably embrittles the alloy. The presence of acicular grains with high stress concentration factors is responsible for the low ability of the Ti-2.5Al-10Mn alloy to withstand plastic deformation. The analysis of the mean mechanical properties also reveals that the amount of residual pores has a much more remarkable effect on the elongation to failure than on the strength/hardness. In terms of ductility, all the strengthening mechanisms previously mentioned and the residual pores work collaboratively against it. This results in a significant loss of ductility with a progressive addition of more Mn.

**Figure 7:**
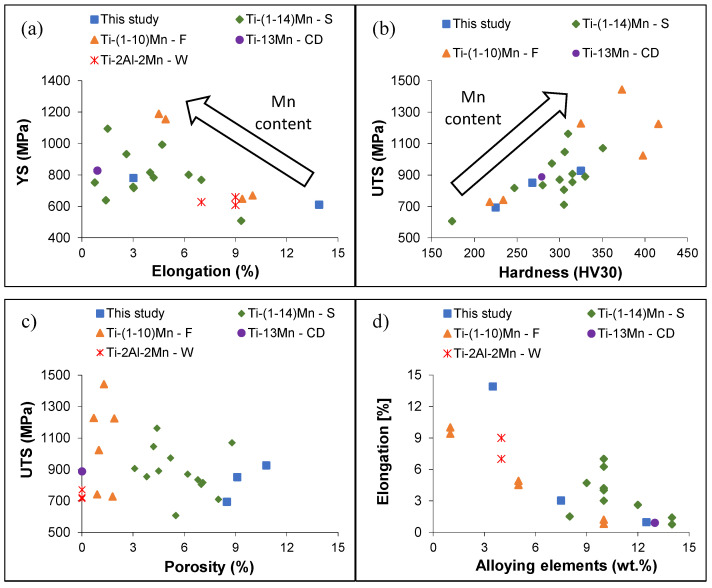
Comparison of the mean mechanical properties of this study’s ternary Ti-2.5Al-xMn alloys with the literature [14,18,20,33,34]: (**a**) YS vs. elongation, (**b**) UTS vs. hardness, (**c**) UTS vs. porosity, and (**d**) elongation vs. amount of alloying elements.

Figure 7 shows a comparison of the tensile behaviour of this study’s ternary Ti-2.5Al-xMn alloys with relevant data found in the literature for sintered Ti-Mn alloys [14,20,33], forged Ti-Mn alloys [20,34], a cold-rolled Ti-13Mn alloy [15], and welded Ti-Al-Mn alloys [18]. As shown in Figure 7a, as the value of YS increases, the elongation decreases due to the strengthening effects brought about the addition of Al and Mn. The Ti-2.5Al-1Mn alloy has better ductility and similar YS to some of the sintered Ti-Mn alloys and the welded Ti-Al-Mn alloys.

The Ti-2.5Al-5Mn alloy has better ductility and similar YS to some of the sintered Ti-Mn alloys and the cold-rolled Ti-13Mn alloy. Differences between the alloys are imputable to the specific amounts of porosity and alloying elements.

As shown in Figure 7b, increases in the amounts of alloying elements, especially Mn, lead to an almost linear increase in both UTS and hardness. For comparable amounts of alloying elements, the UTS/hardness pairs of the Ti-2.5Al-xMn alloys of this study are similar to other sintered Ti-Mn alloys and the cold-rolled Ti-13Mn alloy. However, they are lower with respect to the forged Ti-Mn alloys due to the presence of residual pores.

From the analysis of UTS versus the amount of porosity (Figure 7c), the Ti-2.5Al-xMn alloys of this study have comparable strength values to most of the other alloys considered, despite the greater amount of porosity. This means that even higher UTS values can be achieved if the amount of porosity of the alloys is reduced via thermomechanical processing (e.g., forging or rolling) [35] or via hot isostatic pressing [36]. With respect to the elongation to failure as a function of the amounts of alloying elements (Figure 7d), ductility decreases with higher contents of alloying elements. The Ti-2.5Al-1Mn alloy has the highest elongation value, which is better than that of the sintered Ti-Mn alloys with a lower amount of alloying elements and that of the fully dense welded Ti-Al-Mn alloys.

## 5. Conclusions

This study analysed the processing and properties of a series of Ti-2.5Al-xMn alloys (x = 1, 5, and 10 wt.%). The alloys were manufactured through the simple press-and-sinter blended elemental powder metallurgy approach. Based on the analysis of the results, it can be concluded that the addition of the alloying elements decreases the compressibility of the powder blends. Therefore, the amount of residual porosity in the green and sintered samples increases with the amounts of alloying elements.

However, these values are comparable to those of Ti-based alloys obtained via powder metallurgy processing. The progressive addition of Mn initially refines the typical lamellar microstructure, which eventually transforms into an equiaxed β-grain structure with a needle-like α lamellae. The equilibrium α and β phases are the only ones detected in the alloys.

The addition of the alloying elements, thus, results in a higher amount of stabilised beta, refined microstructural features, greater solid solution strengthening, and higher porosity levels. Consequently, strength and hardness continuously increase, and ductility exponentially decreases. This highlights that the strengthening mechanisms control the resistance to plastic deformation, and porosity greatly affects the ductility of the alloys.

## Figures and Tables

**Table 1:** Details of the raw materials as per the suppliers’ specifications.

Material	Max. Particle Size	Purity	Supplier
Ti	<75 μm (200 mesh)	>99.4%	Goodfellow Ltd. (Cambridge, UK)
Al	<45 μm (325 mesh)	>99.7%	Ecka Granules (Velden, Germany)
Mn	<63 μm (230 mesh)	>99.7%	Sigma-Aldrich (St. Louis, MO, USA)

## Data Availability

All metadata pertaining to this work will be made available upon reasonable requests.
